# Continuous Human Action Recognition Using Depth-MHI-HOG and a Spotter Model

**DOI:** 10.3390/s150305197

**Published:** 2015-03-03

**Authors:** Hyukmin Eum, Changyong Yoon, Heejin Lee, Mignon Park

**Affiliations:** 1 School of Electrical and Electronic Engineering, Yonsei University, 134 Shinchon-Dong, Seodaemun-Gu, Seoul 120-749, Korea; E-Mail: hmeum@yonsei.ac.kr; 2 Department of Electrical Engineering, Suwon Science College, Hwaseong 445-742, Korea; E-Mail: cyyoon@ssc.ac.kr; 3 Department of Electrical, Electronic and Control Engineering, Hankyong National University, Anseong 456-749, Korea; E-Mail: lhjin@hknu.ac.kr

**Keywords:** continuous human action recognition, depth-MHI-HOG (DMH), hidden Markov model, action modeling, action spotting, spotter model

## Abstract

In this paper, we propose a new method for spotting and recognizing continuous human actions using a vision sensor. The method is comprised of depth-MHI-HOG (DMH), action modeling, action spotting, and recognition. First, to effectively separate the foreground from background, we propose a method called DMH. It includes a standard structure for segmenting images and extracting features by using depth information, MHI, and HOG. Second, action modeling is performed to model various actions using extracted features. The modeling of actions is performed by creating sequences of actions through k-means clustering; these sequences constitute HMM input. Third, a method of action spotting is proposed to filter meaningless actions from continuous actions and to identify precise start and end points of actions. By employing the spotter model, the proposed method improves action recognition performance. Finally, the proposed method recognizes actions based on start and end points. We evaluate recognition performance by employing the proposed method to obtain and compare probabilities by applying input sequences in action models and the spotter model. Through various experiments, we demonstrate that the proposed method is efficient for recognizing continuous human actions in real environments.

## 1. Introduction

In everyday life, people exchange information by using language and nonverbal expressions. For example, a person may greet someone by waving a hand. Therefore, it is important to study recognition of nonverbal expressions in order to establish a natural interface. Thus, new studies have been actively researched to recognize speech [1] and nonverbal expressions [2] for exchanging information.

Nonverbal expressions are demonstrated through facial expressions, the gaze, hand gestures, gait, bodily actions, and so on. Specific aspects of subjects are recognized through these expressions [3,4,5,6,7,8], while subject actions likewise use parts of the whole body (the head, arms, legs, *etc.*). It is therefore difficult to automatically recognize actions because people have complex joint structures.

Many systems that recognize nonverbal expressions currently exist. However, most methods recognize only a specific aspect of a subject. A method for recognizing the whole body is relatively rare. Many problems occur when recognizing the whole body instead of a specific portion. Therefore, research has continued to progress in terms of developing and improving whole body recognition.

Actions have been initially recognized using displacement values obtained by attaching sensors to the joints of the body [9]. However, this method is cumbersome and complicated because the sensors are attached to the body. Moreover, it is difficult to calibrate the system because the devices are not always attached to the same area. In addition, it is impossible for the body to engage in natural actions because of the connected cables. Consequently, this method is rarely used.

A similar method proposed in [10] recognizes action through a camera after optical markers are attached to the body. However, in this method, the equipment used is expensive and the creation of a test environment is cumbersome.

Alternative methods exist, such as the vision-based recognition method [11,12], which analyzes continuous human action information with only a camera. This method is not constrained in terms of experimental mechanisms and it apparently provides good recognition results. This approach is less expensive than other methods because only a camera is used; there is no need to attach a device and real-time experiments are possible because the test environment is invariable. For this reason, we recognize continuous human action based on the camera.

In this paper, we use camera sensor that includes an IR emitter and IR depth sensor. The IR emitter (IR projector) emits an infrared light pattern and the IR depth sensor (IR camera) reads and analyzes the infrared beams reflected back to the sensor. The reflected infrared beams are converted into depth information. This information can indicate the distance between an object and the sensor and is used in applications that implement a variety of functions such as object detection, motion tracking, and so on.

In addition, we use a method that calculates the structure through the reflected beams based on the specific light pattern unlike the time of flight (TOF) method. The TOF method measures the time difference between the pulses that are emitted and reflected, and is used in radar and ultrasonic sensors. TOF based camera is method to analyze the phase difference of the reflected wave from the sensor and is used in the outside environment. However, the TOF method requires integration time to remove noise and generates problems. The problems relatively cause lower frame rate, motion blur phenomenon, distance errors, and so on. Therefore, specific light pattern method has the better performance than TOF method but limitation is that it can be used indoors only.

Among several methods used in this paper, two types—template-based feature extraction [13] and the state-space-based model [14]—are explained as related method. We use templates as features and recognize action through modeling the state space.

Template-based feature extraction uses a spatio-temporal template and assumes that a specific pattern is followed. This method easily creates the template and is useful in a fixed environment. The template-based method can representatively describe the motion energy image (MEI) and motion history image (MHI) [15,16]. Bobick and Davis proposed MEI and MHI for human action recognition [17]. It includes prior information and gradually gathers the most recent information. It therefore can readily handle moving objects, such as human action, gait, moving cars, and gestures. In addition, it is easy to create template because MEI and MHI are composed of a simple algorithm.

The modeling method based on state space uses a model that stochastically changes the internal state in accordance with time through input symbols. States are connected to other states, transferred to other states through any probabilities (transitions), and can return to oneself (self-transitions). The hidden Markov model (HMM) is a typical state-space-based method [18,19,20]. Because this method stochastically handles signal variation, it can naturally model spatio-temporal information. It has been applied in various fields because of its efficient and effective algorithm for learning and recognition. The algorithm is particularly renowned for use in speech recognition and online handwriting recognition; furthermore, it has been used for action recognition, gesture recognition, and so on. HMM has a temporal structure that can naturally represent speech, online handwriting, gestures, actions, and more.

**Figure 1:**
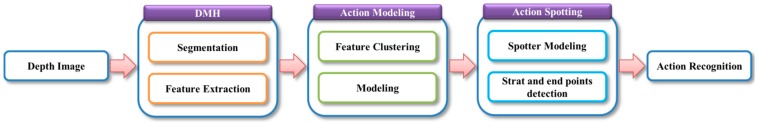
The proposed system process for continuous human action recognition.

Based on these studies, we use MHI because it employs a camera with a fixed background. In addition, we have an interest in spotting to distinguish meaningful action from meaningless action. Therefore, we compose an action model that can be used for recognition, and the spotter model can be employed for spotting. These methods perform the process shown in Figure 1 to recognize a continuous human action.

As shown in the Figure 1, DMH uses the template-based method, action modeling applies the state-space-based method, and action spotting employs the spotter model. In this system, six actions (Bend, Hand, Kick, Run, Walk, and Sit) are recognized by using the process shown in Figure 1. In unexpectedly continuously entered images, DMH features are extracted and action is recognized after detecting the start and end points.

In this paper, continuous actions are continuously performed; for example, these actions (order: Bend → Walk → Kick → Run → other actions) are continuously performed. In other papers [21,22,23], the word “continuous” is generally used. In [21], a person sits down, then stands up, walks forward, bends down to pick up something, and then gets up and walks away. Each of these actions (sitting down, standing up, walking, bending down, and getting up) are primitive actions of a continuous action sequence; however, the transitions between actions are not clearly defined. Therefore, a segmentation method is used to clearly define each of the primitive actions in a continuous action sequence. This approach is typically the same procedure used to find meaningful actions, e.g., the spotting method in this paper.

Our contribution is the recognition of continuous action using the spotter model. This method filters meaningless action and extracts meaningful action from a continuous sequence. Consequently, the start and end points are found automatically. This method is used in online handwriting recognition and in gesture recognition to detect start and end points and thus improve recognition accuracy.

The reason for using the spotter model is that there is a limit to expressing meaningless action because of the wide range of actions. Therefore, we create the spotter model using the information of the action model.

Two examples of meaningless actions are ambiguous and similar actions, and these actions are filtered by the spotting method. In the first case, the ambiguous parts between meaningful actions (Walk and Sit) are removed. In the second case, the Run action is performed after the Bend action. However, similarity to the Walk action is also identified. It is necessary to filter these actions to improve recognition accuracy.

In this paper, we propose continuous human action recognition using the DMH feature and HMM-based spotter model, and we evaluate the performance of this model. The remainder of this paper is organized as follows: Section 2 reviews related work, and, in Section 3, the details of human action spotting and recognition are explained. Experimental results of our system are provided in Section 4. Our conclusions and future plans are described in Section 5.

## 2. Related Work

Camera-based action recognition methods are divided into 3D methods [24,25,26,27,28] and 2D methods [29,30,31,32]. 3D methods perform recognition using 3D modeling of the human body; however, it is difficult to match and model a high degree of freedom in human body joints with them. On the other hand, 2D methods perform recognition using a silhouette appearance of the image as seen by the camera. Because we employ a fixed indoor background, the complex process of creating a skeleton in the 3D method is unnecessary. Therefore, we use the 2D method, which is simpler and easier to use than the 3D method.

Moreover, we use a template method that achieves excellent results when using a fixed background. Although it is difficult to use with changing backgrounds or differing viewpoints, it provides good performance with a fixed background. In addition, human action is not shown in a single image; rather, it appears in a series of images. For these reasons, we employ this method.

To recognize continuous human action, a technique that can extract meaningful action and remove meaningless action is required. This technique is called pattern spotting. As a method for detecting start and end points in human action recognition, pattern spotting can be regarded as a single application. In this paper, action recognition using action pattern extraction is called action spotting and is performed by using the spotter model.

The pattern spotting method is used for online handwriting recognition [33], gesture recognition [34,35], and other purposes; this method affects recognition accuracy. An example of this is included in [34,35]. A spotting method for gesture recognition did not be used in [34], but this technique did be used in [35], and the experimental recognition accuracy of [35] was higher than that of [34]. As a result, the spotting method was found to increase recognition accuracy.

Most of the existing spotting methods use a backward approach. This approach first finds an end point and traces a start point back through an optimal path. Thus, an inevitable time delay occurs, and is not suitable for continuous action recognition.

To solve this problem, we use a forward spotting approach. This approach is similar to that used in [10], but without a sliding window and accumulative HMM methods, as they can cause delays. Therefore, we propose using a spotter model that finds start and end points through the flow of action changing with time to recognize actions.

## 3. Proposed Method

**Figure 2:**
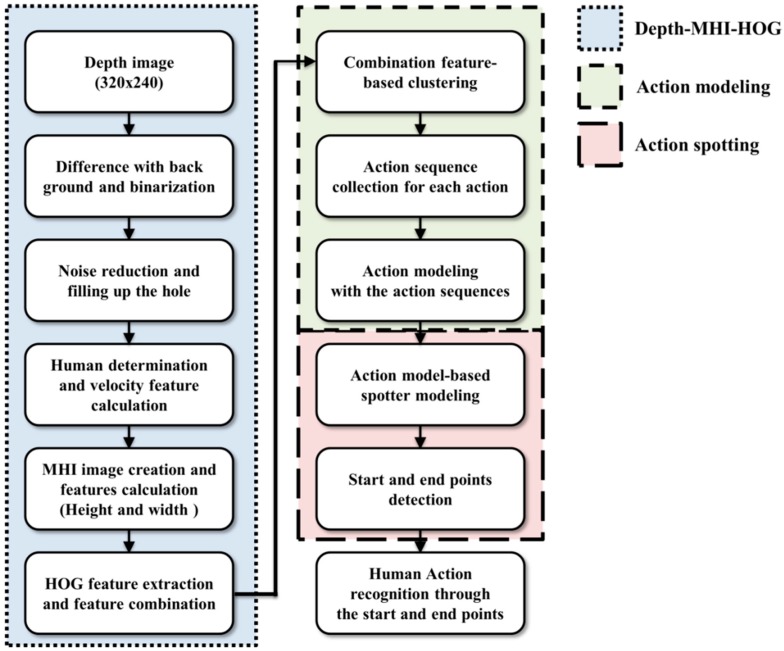
Block diagram of system for human action spotting and recognition.

The present objective is to recognize six actions of the human whole body image based on depth information obtained with a fixed camera. The proposed spotting approach and action recognition method are divided into four components: DMH, action modeling, action spotting, and recognition. Details of the proposed system are illustrated in Figure 2.

First, DMH performs segmentation and feature extraction. The main goal of image segmentation is to separate the foreground and background and to remove noise from an image, which eliminates the background, by using a filter. This preprocessing identifies an object through the depth image received from a camera. We employ a simple method of background subtraction using the depth image, weighted binarization, and a median filter for image segmentation. Moreover, feature extraction reduces the computational cost of recognition because features of the segmented object are used without a full image. The features we use include MHI and HOG [36], which are more robust than color information in changing light and clothes on account of employing depth information. In addition, the method is easier to handle than methods using human skeletons or background models and quickly extracts objects.

Second, action modeling is used to model various human actions through extracted features. The input features are converted to human action sequences by employing k-means clustering based on features; action is then modeled using clusters as HMM input. Because human action is shown by continuous sequences, we use k-means clustering in the action modeling. Feature clustering is included in [31,32] and other papers use a codebook created by vector quantization. A code book generation and symbol selection step are used in [31,32], and this step creates an action sequence. However, in this procedure, we chose to use k-means clustering because this method is easily implemented and has good performance among clustering methods to easily separate a large amount of data. In addition, to select the action that is stochastically the most similar to an action sequence, human action is modeled. Action modeling can be used to create models of meaningful actions through HMM rather than labeling, which means that transition and output probabilities are trained using HMM. Because action changes with time, we use HMM. HMM naturally models temporal and spatial information because the variation of the signal is stochastically handled. In summary, HMM has a temporal and spatial structure, and is able to stochastically model the correlation between frames. Therefore, HMM is more appropriate for continuous action recognition. Moreover, we subdivide six actions into twelve actions to improve recognition accuracy before the action is modeled.

Third, meaningless actions are filtered from continuous actions, and action start and end points can then be detected in action spotting. This comprises the created models in the action modeling step; the spotter model is then modeled. The spotter modeling procedure creates a new model using the properties of action models made by HMM. HMM has implicitly separate attributes. These attributes represents that each states and self-transition express the sub patterns of action pattern and transition to other state indicates the combined ordering of the sub patterns in action pattern. Transition probability includes self-transition and transitions to another state. Transition and output probabilities are modified, and these modifications of probabilities apply to the spotter model and consist of a new structure. Action spotting serves to find meaningful action and is used to detect and recognize the precise action by means of the action start and end points.

Finally, human actions are recognized using the start and end points obtained through the spotter model. The evaluation method of the recognition acquires probabilities of each of the action and spotter models based on input sequences; the results are then compared. The probabilities of action models should be higher than that of the spotter model. Additionally, the highest action is recognized among the actions that have the higher probabilities. The process for recognizing meaningful action uses the previously modeled models in accordance with the input sequences. Furthermore, the twelve actions in action modeling, which were subdivided for accuracy, are combined as six actions; the action is then recognized.

The following are some assumptions for recognizing human actions in our system: an indoor image of human action is employed; depth information is applied; a fixed camera is used; and the action of a single object is recognized.

### 3.1. Depth-MHI-HOG

DMH removes the background based on depth information [37]. It then segments the image through binarization and a filtering procedure, and it extracts features using MHI and HOG. After the object is found by using depth information, MHI is created; features are then extracted using HOG. With DMH, calculations are simple and efficient. Only depth information can be easily used because it employs silhouettes. Moreover, MHI includes much more information than other features because it is comprised of previous information; furthermore, it creates the template. In addition, MHI is often used in action recognition because the principle of the algorithm is simple and easy. HOG is employed to express a histogram as the number of gradient occurrences in the local region. It is a useful algorithm that is often used in object detection.

#### 3.1.1. Segmentation

**Figure 3:**
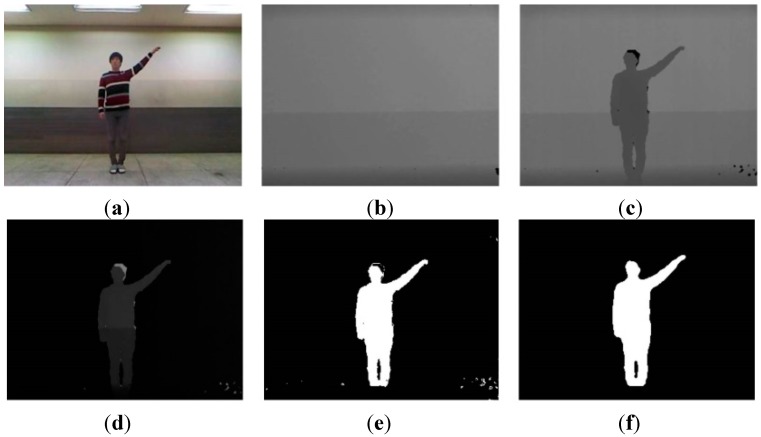
Color image and segmentation process images: (**a**) color image; (**b**) background image; (**c**) depth image; (**d**) difference image; (**e**) weighted binary image; and (**f**) median filter image.

The image segmentation process with depth information is shown in Figure 3. The input image consists of the foreground and background that are to be extracted and removed, respectively. The background is divided into static and dynamic backgrounds. The dynamic background is shown in an outdoor environment, whereas an indoor environment has a predominantly static background.

Because our system recognizes human actions based on indoor images, such as the one shown in Figure 3c, a simple and easy algorithm is used. Background and foreground are efficiently separated using only depth information, such as in Figure 3b,c. In a depth image more than in a color image, the background and foreground can be easily segmented from depth information. For example, the length of clothes may change according to the season, and clothes come in a variety of colors. For these reasons, segmentation by color information is difficult. Further, a comparison of Figure 3a,c shows that light occurs in the color image but not in the depth image.

Typically, the portion of the image with the greatest depth information is the background. However, in the case of fluorescent lighting and a coated ground, noise is reflected by light occurs in the input image. Thus, we first store background depth image; after the input depth image and background image are subtracted, noise is removed by a threshold. Through this process, we identify the foreground. It removes consistently occurring noise; moreover, calculation is fast because the simple difference of depth information is used. The thresholds that are employed as the background are values of the largest and smallest parts. The greatest threshold is typically a background. Moreover, for the present purposes, the smallest threshold serves to remove noise, and a certain distance should be maintained because action is recognized using the whole body. We therefore separate the background by two types of thresholds and identify the foreground as a grayscale image.

To note, the feature extraction process requires a binary image, and therefore binarization is needed. During binarization, unnecessary information is removed as much as possible, while important information should be included; unnecessary information, such as noise, can be included in the foreground. However, because some noise is included in the foreground image, we have chosen the weighted binarization method. A weight value maintains significant information and reduces unnecessary information. The filtering process then removes the remaining unnecessary information. The formula of weighted binarization is expressed as follows:

(1) B(x,y,t) = [D(x,y,t)⋅σ]/255

where B(x,y,t)  is the binary image performed by weighted binarization,  D(x,y,t) is the foreground (grayscale image) obtained by image segmentation, and σ is the weight value. Using this method, important information is maintained and unnecessary information can be partially removed. However, because some noise is still included, we remove it using a median filter. This filter is known to be effective at removing random noise, such as salt noise. It covers the center pixel with a window; the center pixel value is then replaced by the median pixel value within the window. For the present purpose, the window size in the median filter is 5 × 5 pixels. When applying the filter, a hole sometimes occurs in the region of the human. This hole is filled using the region boundary. Finally, we extract features with the binary image obtained in this method.

#### 3.1.2. Feature Extraction

We extract features based on the binary image obtained from preprocessing. Because human actions are not shown in a single image, such as in human detection, facial recognition, or pose recognition, it requires an information series from previous images. We therefore create MHI using an information series to recognize human action. Importance degree exists in the information. Therefore, both important and unimportant information exist among the previous data. As time passes, the important information decreases and is gradually erased; it is not used. However, recently received information has a higher degree of importance.

**Figure 4:**
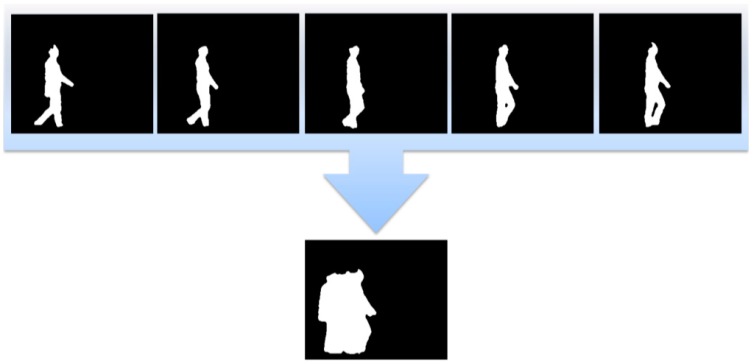
MHI creation processes for walking action.

For example, if a person is walking from left to right, the moving image is gradually received by the camera. At this time, the importance of the early information gradually decreases, which can be expressed as a decreasing value. On the other hand, a newly produced human image on the right is described as a maximum value. Thus, if a person walks or runs, it can be expressed as generating and erasing a silhouette in MHI. MHI commonly consumes less time and is suitable for recognizing human action appearing at a given moment. It employs the following Equation (2):

(2) $$H(x,y,t)=\{\begin{array}{l} \tau \;\;\;\;\;\;\;\;\;\;\;\;\;\;\;\;\;\;\;\;\;\;\;\;\;\;\;\;\;\;\;\;\;\;\;\;\;\;\;\text{if}\;B(x,y,t)=1\\ \max(0,H(x,y,t-1)-\delta )\;\;\;\;\;otherwise \end{array}$$

where H(x,y,t) is the current MHI, H(x,y,t−1) is the previous MHI, B(x,y,t) is the current binary image, τ is the maximum value of importance degree, and δ is the decreasing value (reduction coefficient) of the importance degree. If the pixel value of the current incoming binary image B(x,y,t) is one, the pixel value of MHI is the maximum value. Otherwise (*i.e.*, the pixel value is zero), MHI subtracts the reduction coefficient from the pixel value of the previous MHI; a higher value is then selected after comparing the subtraction value and minimum value (zero). Accordingly, the pixel value where the action is not shown is zero. The MHI creation is shown in Figure 4.

After the MHI creation, features are extracted using HOG. The HOG feature is widely used for detecting the object; Dalal and Triggs proposed this method [38]. In addition, the human detection algorithm was developed by using HOG in the still image.

The HOG method can describe the local area within the image (object appearance and shape) and distribution of the edge orientation (intensity gradient of the light). This method serves to count occurrences of gradient orientation in the local area; values can vary depending on the spatial organization, gradient calculation method, and normalization method. Calculation of this feature divides the image into small areas called cells, which are interconnected. It is then created by forming the gradient orientation or edge orientation for the pixels within the cells. A combination of these histograms represents this feature.

The HOG feature process first calculates the gradient of the input image. A typical method is to apply a one-dimensional discrete differential mask as the horizontal orientation ($D_{X}=[-1\;\;\;0\;\;\;1]$) and vertical orientation ($D_{Y}={\left[-1\;\;\;0\;\;\;1\right]}^{T}$).

Convolution mask of horizontal orientation:$I_{X}=H(x,y,t)*D_{X}$(3)Convolution mask of vertical orientation:$I_{Y}=H(x,y,t)*D_{Y}$(4)Size of gradient:$\left|G\right|\;\;\;=\;\;\;\sqrt{{I_{X}}^{2}+{I_{Y}}^{2}}$(5)Orientation of gradient:$\theta \;\;\;=\;\;\;\arctan\frac{I_{Y}}{I_{X}}$(6)Signed gradient:$\alpha_{Signed}=\{\begin{array}{l} \alpha \;\;\;\;\;\;\;\;\;\;\;\;\;\;\;\;\;\;\;\;\;\alpha \geq 0\\ \alpha +360\;\;\;\;\;\;\;\;\;\alpha <0\; \end{array}$(7)Unsigned gradient:$\alpha_{Unsigned}=\{\begin{array}{l} \alpha \;\;\;\;\;\;\;\;\;\;\;\;\;\;\;\;\;\;\;\;\;\alpha \geq 0\\ \alpha +180\;\;\;\;\;\;\;\;\;\alpha <0\; \end{array}$(8)

When the image is produced, convolution masks of the horizontal and vertical orientations (Equations (3) and (4), respectively) are applied to the image, and the orientation and gradient size are calculated. Second, histograms of the divided cells are calculated. Each pixel value in the cell is calculated as the orientation of the gradient through an advanced gradient calculation. These values are spread on orientation histogram bands, which are set as the number of bins. Cells are comprised of rectangular shapes in the image. As an expression of the gradient, the histogram bands are evenly distributed from 0 to 360 degrees (Equation (7)) or from 0 to 180 degrees (Equation (8)).

**Figure 5:**
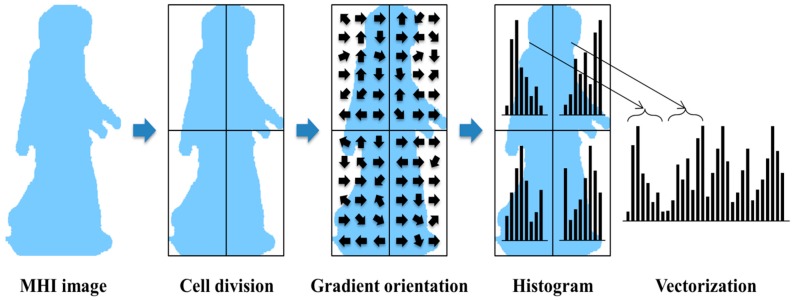
Extraction process of MHI-HOG feature vector.

MHI is received as the input and the HOG feature is then created. This process is shown in Figure 5. In addition, in our system, the number of cells is four (2 × 2) and the number of bins is nine. We therefore create a 36-dimensional vector.

### 3.2. Action Modeling

#### 3.2.1. Feature Clustering

In a clustering method, each vector is grouped as several sets of a feature vector. Among these methods, k-means clustering [39] classifies the feature vectors in the cluster of the closest vector. his method employs the distance between each of the data represented by the vector ($X=[x_{1},\;\;x_{2},\;\;x_{3},\;\;\cdots ,\;\;x_{N}]$). The distance measurement employs the Minkowski distance as the secondary Euclidean distance.

This method can consist of steps such as initialization, data cluster estimation, and the center renewal of a new cluster. First, the initialization decides the number of clusters (k) generated in the data set ($\left[x_{1},\;\;x_{2},\;\;x_{3},\;\;\cdots ,\;\;x_{N}\right]$) and creates an initial center set ($\left[y_{1},\;\;y_{2},\;\;y_{3},\;\;\cdots ,\;\;y_{K}\right]$). The center vector of the initial cluster is randomly chosen among the vectors that belong to the data set. Second, the data cluster estimation step includes data ($x_{n}$) in the cluster ($z_{i}$) if it is close to the center ($y_{i}$). Eventually, the data set is divided (Equation (10)) into the clusters ($\left\{z_{1},\;\;z_{2},\;\;z_{3},\;\;\cdots ,\;\;z_{K}\right\}$) by using the Euclidean distance (Equation (9)):

(9) $$d(x_{n},\;\;y_{i})\;={\left\|x_{n}-y_{i}\right\|}^{2}$$

(10) $$z_{i}=\{x_{n}|d(x_{n},\;\;y_{i})\;\leq \;d(x_{n},\;\;y_{j})\;\}\;\;\;\;\;\;\;\;\;\;where\;j=1,\;\;2,\;3,\;\;\cdots ,\;\;K$$

Third, each center is updated in the new clusters obtained from the data cluster estimation. The new center is calculated as an average value of the data that belongs to each cluster. The convergence of this method is performed by the following two conditions. If one of the conditions is satisfied, it is converged:

(11) $$\;\left|\;y_{j}\;-y_{i\neq j\;}\right|\leq TH_{1}\;\;\;\;\;\;\;\;\;\;where\;j=1,\;\;2,\;\;3,\;\;\cdots ,\;\;K$$

(12) $$\;\left|\;y_{j(t)}\;-y_{j(t-1)\;}\right|\leq TH_{2}\;\;\;\;\;\;\;\;\;\;where\;j=1,\;\;2,\;\;3,\;\;\cdots ,\;\;K$$

Equation (11) is repeated until the distance between the clusters is smaller than the threshold value ($TH_{1}$). When a longer center of the cluster is not changed, Equation (12) is finished; in the case of the changed cluster center, it is repeated. A movement of the center point and change of the cluster depends on the start point of the cluster and number of the repetition.

In the dataset, it is difficult to determine the optimal number of clusters. However, the method we used to choose the k value was based on data quantity and the number of subdivided actions. First, the k value should be higher than the number of subdivided actions. Typically, if the k value is increased, the data are subdivided and the performance is good. However, clustering takes a long time. Therefore, we gradually increased the k value and selected the value needed to reach the appropriate level. Thus, the k value was set to 25.

**Figure 6:**
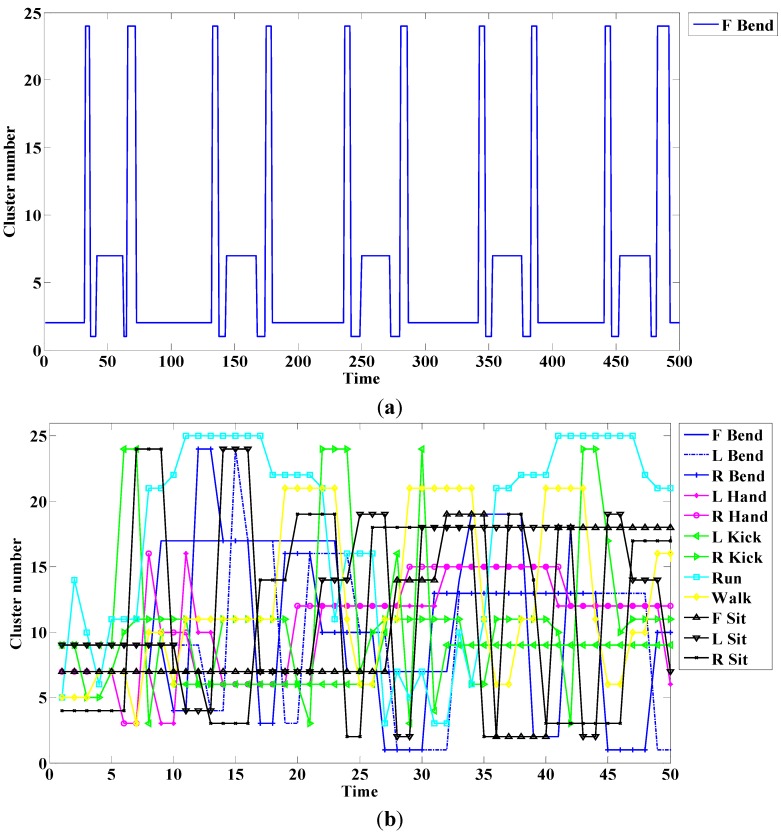
Cluster number for (**a**) a single action; and (**b**) all actions.

An example of this method is shown in Figure 6. The cluster traces for the single action; all actions are respectively shown in Figure 6a,b.

In this case, the front, right, and left are respectively denoted as F, R, and L. As shown in Figure 6a, a periodic trace is indicated if a single action is repeated. Figure 6b lists cluster numbers according to time. We create a sequence of human actions, as in Equation (13), through the cluster; the sequence is used as the model of observation introduced in the next part. A model number is denoted by m and n is the number of sequences. Each sequence data is a cluster number that indicates the cluster trace of the action:

(13) $$S_{m}=[s_{1},\;\;s_{2},\;\;s_{3},\;\;\cdots ,\;\;s_{n}]$$

#### 3.2.2. Modeling

We create models to recognize human actions based on HMM [19]. The HMM method has a temporal structure and classifies continuous patterns in a statistical manner. It can express action through the feature vector and uses observation according to time. We use the feature vector obtained from DMH. The feature vector is then changed to one of the cluster numbers using k-means clustering because of HMM. It is symbolized and uses an input of HMM.

**Figure 7:**
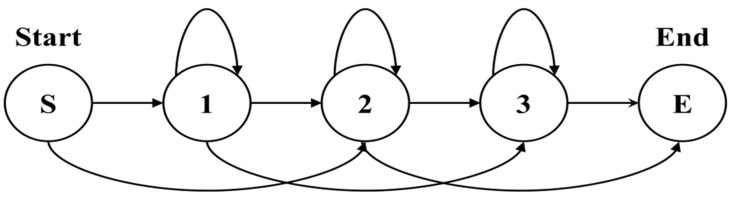
Example of the left-right model.

HMM chooses the structure that depends on the amount of training data, the model to represent, and the recognition target. The left-right (L-R) model has many applications because human action is essential for the temporal sequence to be considered. In this model, the state number advances with the passing of time or proceeds in an increasing direction. Because the state transfers from left to right, it is an L-R model as Figure 7.

HMM is expressed by λ={A,  B,  π}, where A is the matrix of probability $a_{ij}$ that transfers state $s_{i}$ to state $s_{j}$, B is the matrix of probability $b_{j}(k)$ that observes a symbol k in state $s_{j}$, and $\pi_{i}$ is the initial probability started in state $s_{i}$. If there are n number of states ($Q=\left\{q_{1},\;\;q_{2},\;\;q_{3},\;\;\cdots ,\;\;\;q_{n}\right\}$) and m number of observation symbols ($V=\left\{v_{1},\;\;v_{2},\;\;v_{3},\;\;\cdots ,\;\;\;v_{m}\right\}$), this can be expressed as follows:

(14) $$A=\left\{a_{ij}|a_{ij}=P(q_{t+1}=S_{j}|q_{t}=S_{i})\right\}\;\;\;\;\;\;\;\;\;\;where\;\;\;1\;\;\leq \;\;i,\;\;j\;<\;n$$

(15) $$B=\left\{b_{j}(k)|b_{j}(k)=P(O_{t}=v_{k}|q_{t}=S_{j})\right\}\;\;\;\;\;\;\;\;\;\;where\;\;\;1\;\;\leq \;\;k\;\;<\;m$$

(16) $$\pi =\left\{\pi_{i}|\pi_{i}=P(q_{1}=S_{i})\right\}\;\;\;\;\;\;\;\;\;\;where\;\;\;1\;\;\leq \;\;i\;\;<\;n$$

The *n*-by-*n* transition probability is Equation (14), the *m*-by-*n* observation probability is Equation (15), and the initial probability is Equation (16). The probability of finding the observation sequence is P(O|λ). Each human action model calculates the optimal λ to maximize the likelihood with respect to the observation sequence using the Baum-Welch algorithm. It is calculated by the observation sequence ($O=\left\{o_{1},\;\;o_{2},\;\;o_{3},\;\;\cdots ,\;\;\;o_{T}\right\}$) and the following equations.

(17) $$\begin{array}{l} \xi_{t}(i,j)=P(q_{t}=S_{i},\;q_{t+1}=S_{j}\;|O,\;\;\lambda )\\ \;\;\;\;\;\;\;\;\;\;\;\;\;\;\;=\;\frac{P(q_{t}=S_{i},\;q_{t+1}=S_{j},\;O|\;\;\lambda )}{P(O|\;\;\lambda )}\\ \;\;\;\;\;\;\;\;\;\;\;\;\;\;\;=\;\frac{\alpha_{t}(i)a_{ij}b_{j}(o_{t+1})\beta_{t+1}(j)}{P(O|\;\;\lambda )}\\ \;\;\;\;\;\;\;\;\;\;\;\;\;\;\;=\;\frac{\alpha_{t}(i)a_{ij}b_{j}(o_{t+1})\beta_{t+1}(j)}{\sum_{i=1}^{n}\sum_{j=1}^{n}\alpha_{t}(i)a_{ij}b_{j}(o_{t+1})\beta_{t+1}(j)} \end{array}$$

(18) $$\gamma_{t}(i)=P(q_{t}=S_{i}|O)=\frac{\alpha_{t}(i)\beta_{t}(i)}{P(O|\lambda )}=\sum_{j=1}^{n}\xi_{t}(i,j)$$

where $\xi_{t}(i,j)$ is the probability that retains state $s_{i}$ at time t and state $s_{j}$ at time t+1. $\gamma_{t}(i)$ is the probability that exists in state $s_{i}$ at time t when model λ and observation sequence O are given. $\;\alpha_{t}(i)$ and $\beta_{t}(i)$ are forward and backward variables, respectively. This means that the observation probability for a partial state when state $s_{i}$ exists at time t and the action model (Equations (19)–(21)) is estimated by using Equations (17) and (18) as follows:

(19) $$\bar{\pi_{i}}=\gamma_{1}(i)$$

(20) $$\bar{a_{ij}}=\frac{\sum_{t=1}^{T-1}\xi_{t}(i,j)}{\sum_{t=1}^{T-1}\gamma_{t}(i)}$$

(21) $$\bar{b_{j}(k)}=\frac{\sum_{t=1,o_{t}=v_{k}}^{T}\gamma_{t}(i)}{\sum_{t=1}^{T}\gamma_{t}(i)}$$

Moreover, the action model is influenced by the number of states. After the structure is chosen, the number of states of the model is decided. It is changed by the complexity of the model. We choose the number of states depending on the following procedures and Box 1:

(1) Initialization: The number of states of the model is set to one.
(2) Training: The model is trained and created using the Baum-Welch algorithm.
(3) Evaluation: The trained model is evaluated by employing the Viterbi algorithm; the likelihood value is then stored.
(4) Increase and repetition: The number of states is increased until N; then Steps 2 and 3 are repeated.
(5) Selection: The stored likelihood values are compared; a model of the maximum likelihood value is then selected.

Box 1 **Action model selection according to the number of states.**

For *i* = 1:*N*

Step 1: Determine {*A_i_*, *B_i_*}, according to the number of states.
Step 2: Generate *M_i_* = *BW* (*A_i_*, *B_i_*, *Seq*) by the Baum-Welch algorithm.
Step 3: Calculate and store *L_i_* = *VTB* (*M_i_*, *Seq*) by the Viterbi algorithm.

End.

Step 4: Find the index of the maximizing likelihood $[idx,val]=\max_{i}\;L_{i}$.

Step 5: Select *M_idx_* about the index.

Here, N is the maximum number of states, $A_{i}$ is the transition probability, $B_{i}$ is the output probability,  Seq is the human action sequence, and $M_{i}$ is the action model. In addition, $L_{i}$ is the likelihood, val is the maximum value, idx is the index of the maximum value, $M_{idx}$ is the action model for the index of the maximum value, BW is the Baum-Welch function, and VTB is the Viterbi function [18].

### 3.3. Action Spotting

#### 3.3.1. Spotter Modeling

The spotter model [40,41,42] is required for automatically dividing actions to be recognized from all other actions. We divide meaningful and meaningless actions. The spotter model is used to extract a meaningful action that appears while we continuously observe actions; it identifies the start and end points for recognizing action. That is, it serves as an indicator of start and end points of action and as a criterion of recognition.

However, there is a limit to expressing meaningless action because of the wide range of actions. In pattern spotting, the core model for known patterns and a garbage model for unknown patterns are commonly defined. At this time, the garbage model is trained by using unknown data of a finite set. However, because meaningless action patterns cannot be defined in action spotting, a garbage model that expresses meaningless patterns through training cannot be used. Therefore, we apply the states of the trained action model by employing implicitly separated attributes; moreover, we introduce a new model to determine the degree of matching results in the action model. We implicitly separate the attributes so that the states and transitions implicitly express order and sub patterns of action patterns, respectively, in the trained HMM. In addition, we construct another model that can be matched with a new pattern created by changing the combined ordering of action sub patterns through the attributes; this is the spotter model.

The structure of this model is formed by implicitly separating the attributes with ergodic forms because the model should express all actions, including the core action. The ergodic model is a fully connected model that can be reached by a single transition from each state to all other states. It is useful for constructing close matches for all combination results.

**Figure 8:**
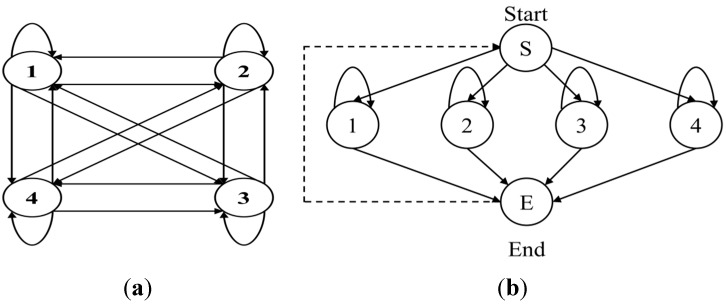
Ergodic model structure of HMM: (**a**) fully connected structure; (**b**) simply connected structure.

To simplify the model, we introduce the null state and null transition, which respectively mean that the observation symbol is not handled and the transition probability is not calculated. Accordingly, the process of creating the spotter model is outlined in Figure 8b and in the following procedures and Box 2.

(1) Output probabilities: Output probabilities of all action models are inserted as output probabilities of all states between the null (start and end) states included in the model.
(2) Transition probabilities from the start to index state: These probabilities are uniformly divided according to the number of states.
(3) Self-transition probabilities in index states: Self-transition probabilities of action models are used.
(4) Transition probabilities from the index to end state: It uses values to subtract the self-transition probability from the total probability (one minus self-transition).
(5) Transition probability from the end to start state. It is immediately transferred on account of the null state.

Box 2 **Spotter model creation process.**

Step 1: Designate *S_s_* and *S_e_* as the null state {*S_s_*, *S_e_*} = *Null* and set the spotter state as *S* = {*S_s_*, *S_i_*, *S_e_*}.

Step 2: Copy the output probability of the action model into the index state *B*(*S_i_*) = *B*(*M*).

Step 3: Equally determine the transition probability of *S_i_* from *S_s_* according to the number of states of all action models $a_{si}=\frac{1}{N_{M}}$.

Step 4: Duplicate the self-transition probability of action model *a_ii_* = *a_M_ii_*.

Step 5: Create the transition probability of *S_e_* from *S_i_* by subtracting the self-transition probability from the whole probability *a_ie_* = 1 − *a_ii_*.

Step 6: Directly transfer *S_e_* to *S_s_*; *a_es_* = 1.

Here, $S=\{S_{s},\;\;S_{i},\;\;S_{e}\}$ is the spotter state (start, index, and end states), M denotes the action model, $B(S_{i})$ is the output probability of the index state, B(M) is the output probability of the action model, and $a_{si}$ is the transition probability from the start state to the index state. In addition, $N_{M}$ is the total number of states in the action model, $a_{ii}$ is the self-transition probability of the spotter model, $a_{M\_ii}$ is the self-transition probability of the action model, $a_{ie}$ is the transition probability from the index state to the end state, and $a_{es}$ is the transition probability from the end state to the start state.

#### 3.3.2. Start and End Point Detection

We detect start and end points [40] based on the spotter model obtained from the above method. It does not include meaningless action when recognizing action and it affects recognition accuracy. This method is performed using evaluation values of all (action and spotter) models after obtaining action data through the DMH features and k-means clustering.

First, the likelihood is calculated by using each action data as input in the models; then, the likelihood of the action and spotter models is compared. If the likelihood of the spotter model is higher than that of the action model, it is determined that an action has not begun. In the opposite case (Equation (22)), our system observes whether the action continuously proceeds; action may have begun and would comprise the precondition:

(22) $$P(X|\lambda_{Action})>P(X|\lambda_{Spotter})$$

If the likelihood of the action model is continuously higher than that of the spotter model, it is considered an action and the end time is awaited. However, if the taking of insufficient time is determined as noise, the start point is again identified. We use this method for detecting start and end points because the likelihood of the action model is higher than that of the spotter model in the case of the beginning of meaningful action. In this part, the spotter model is the criteria for finding start and end points; it can be described as a threshold. We detect start and end points through the following procedures and Box 3:

(1) Precondition: The likelihood of the action model is higher than that of the spotter model, as expressed in Equation (22).
(2) Removal and repetition: If Step 1 is repeated for insufficient time, the first point is removed and Step 1 is then repeated.
(3) Start point detection: If Step 1 is repeated for sufficient time, the first point is determined to be the start point.
(4) End point detection: The last point is determined to be the end point if the likelihood of the spotter model is less than that of the action model and overlapping points do not exist.
(5) Repetition: Action is recognized by these points and models; then, Steps 1 through 4 are repeated.

Box 3 **Box 3. Start and end point detection process.**

For *t* = 1:*T*

Step 1: Find a point (*t*) that satisfies the precondition.
Step 2: Remove the point (*t*) that is repeated for insufficient time.
Step 3: Determine the start point that is repeated for sufficient time.
Step 4: Decide the end point that satisfies the condition of Step 4.
Step 5: Recognize the action through the corresponding sequence from start to end points; then, repeat Steps 1 through 4.

End.

**Figure 9:**
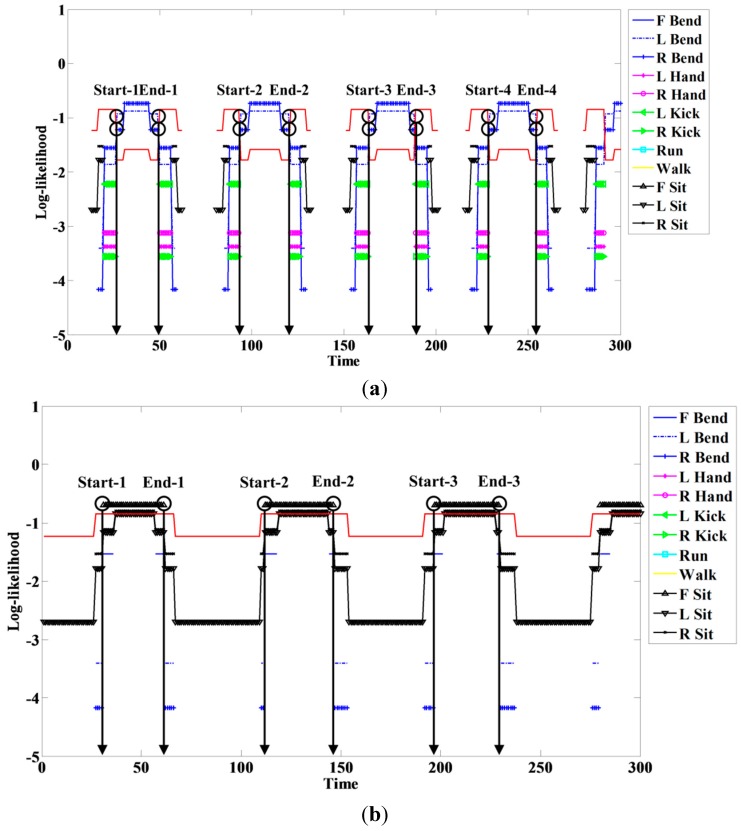
Start and end point detection for (**a**) R Bend; and (**b**) F Sit.

In this case, t is the current time or point and T denotes total time. We detect start and end points using this method. An example of start and end point detection using this method is shown in Figure 9.

**Figure 10:**
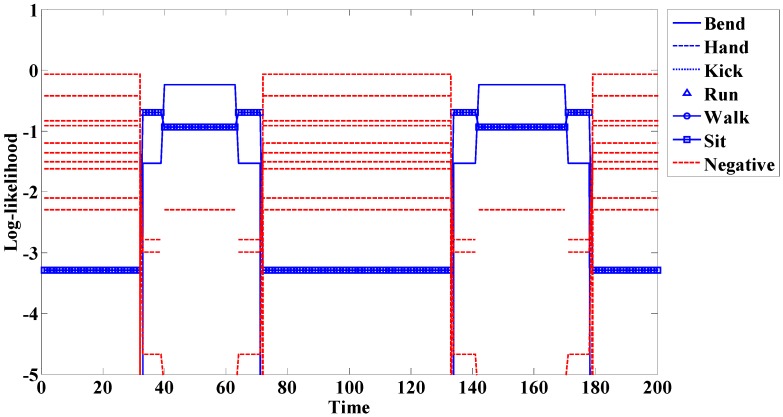
Example of action recognition without the spotting method.

If the spotting method is not used, the problem seen in Figure 10 occurs. In this experiment, we do not use the spotting method and add 10 negative actions. Figure 10 represents the Bend action experiment. The transition before and after the meaningful action (Bend: 40–63 times, 142–170 times) is similar to another action (Sit: 33–39 times, 64–71 times, 134–141 times, 171–178 times). Misrecognition occurs because of this similarity. Furthermore, because this system adds more models, additional time was needed compared to the spotter model. This system is also larger than that of the spotter model. When these problems are removed, accuracy is increased. Thus, it is necessary to use the spotting method to filter meaningless actions.

In addition, there is a limit to expressing meaningless action because of the wide range of actions. However, a wide range of meaningless actions may be included because the use of the spotter model as described above means that the remainder subtracted by meaningful actions is considered. We recognize action using the action models and spotter model obtained by these methods. The start and end points are detected and actions are recognized through the comparison of action models.

### 3.4. Action Recognition

**Figure 11:**
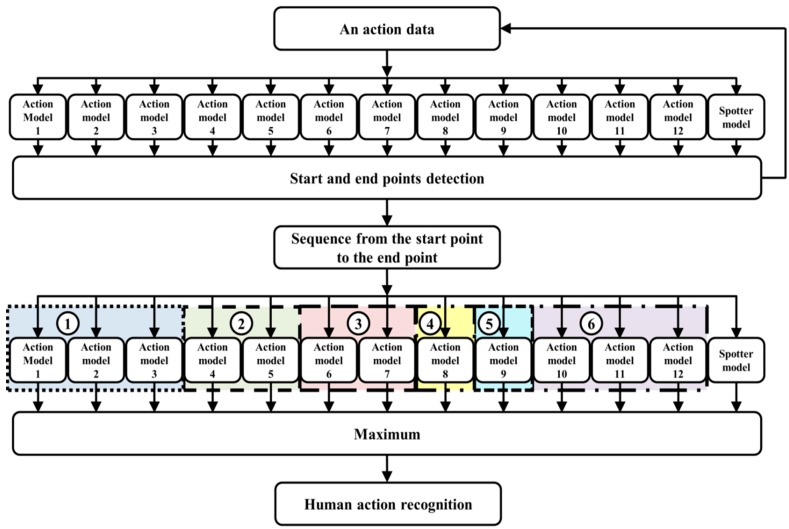
The proposed method for human action recognition.

The process of action recognition is shown in Figure 11. It is based on action models and the spotter model. First, start and end points are detected by all action models and the spotter model after action data is continuously received. The action is then evaluated and recognized using the detected start and end points. Additionally, we analyze the twelve subdivided models by considering the direction of some actions for improving accuracy. We then again combine the subdivided models during recognition. As a result, six actions are recognized using this method.

**Figure 12:**
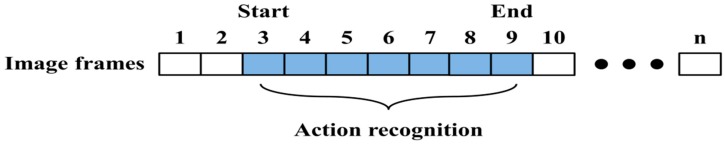
Action recognition for start and end points of image frames.

We create the spotter model to recognize meaningful action, as shown in Figure 11. We then detect the start and end points based on this model. From these points, the input sequence is evaluated using the action models and spotter model. During recognition, the spotter model is used as the threshold, similar to the start and end points. An example of start and end points detection and action recognition among continuous image frames is shown in Figure 12.

The input of symbol sequence Y is created by using the start and end points and probability for each of the action models ($\lambda_{i}$); this is represented as follows:

(23) $$P(Y|\lambda_{i})=\;\sum_{i=1}^{N}\sum_{j=1}^{N}\alpha_{t}(i)a_{ij}b_{j}(y_{t+1})\beta_{t+1}(j)$$

It is evident that the model provides the likelihood and maximum value. When start and end frames are compared, the likelihood of the action model to be recognized is gradually higher than that of other models; this is shown in the experimental results. At the end of the frame, the model that has the highest likelihood is determined to be the recognition result.

## 4. Experimental Results

### 4.1. Experimental Environment

In our evaluation, depth information of 640 × 480 pixel resolution was received at 30 fps using IR projector and IR camera sensor, and distance of the sensors was 7.5 cm. The field of view of camera was vertically 43 degrees and horizontally 57 degrees. These sensors extract depth information using a method that reads the infrared pattern based on triangulation and disparity is used for acquiring the depth information. The computer had an Intel Core i5-3570 3.4 GHz processor and Windows 7 operating system. The distance between the camera and subject was approximately 4 to 5 m.

Actions were divided into the categories of Bend, Hand, Kick, Run, Walk, and Sit. The Bend, Hand, and Kick actions were subdivided according to the direction of the actions; these are shown in Table 1. Accordingly, the total number of actions was twelve. However, these actions were again combined into six types in the action recognition process.

**Table 1:** Images and subdivision of actions.

Action	Subdivision	Images	Action	Subdivision	Images
Bend	F Bend	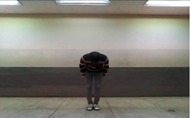	Sit	F Sit	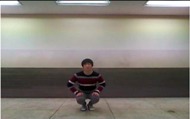
	L Bend	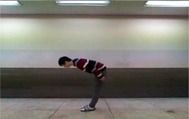		L Sit	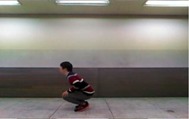
	R Bend	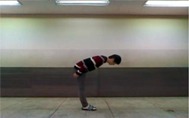		R Sit	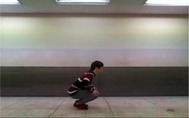
Hand	L Hand	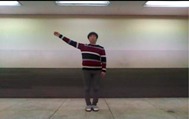	Kick	L Kick	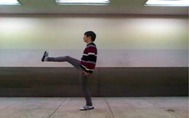
	R Hand	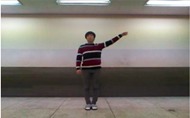		R Kick	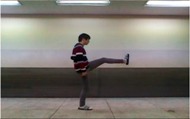
Walk	Walk	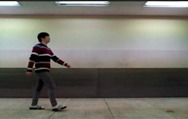	Run	Run	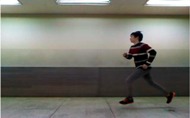

Twelve types of continuous single actions were evaluated using approximately 3000 to 5000 image frames. Additionally, various continuously performed actions were evaluated using approximately 2000 image frames. In the action model learning process, input image frames for each action were converted to observation sequences; the models then learned by these sequences.

Table 1 depicts the subdivided images used in the experiment; for improved comprehension, color images were used. In the action modeling process, the twelve subdivided actions were modeled, and six actions were recognized by their combination in the recognition process. This method was used to improve recognition accuracy.

### 4.2. Segmentation

**Figure 13:**
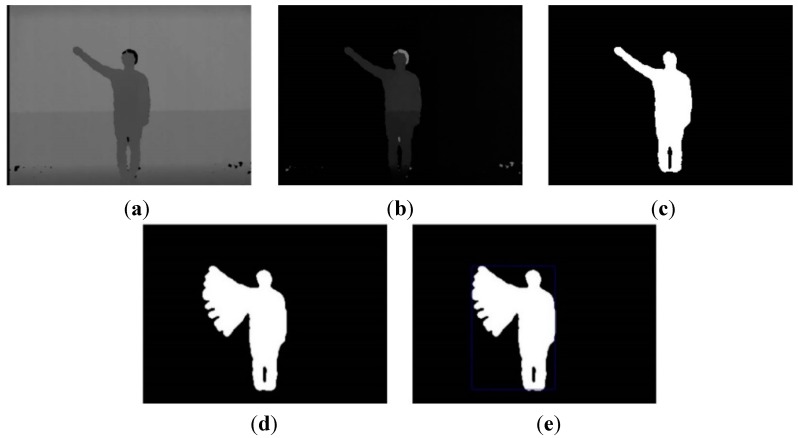
Example of handing action detection process: (**a**) depth image; (**b**) difference image; (**c**) median filter image; (**d**) MHI image; (**e**) detection image.

To locate the target, we segmented the foreground and background. As shown in Figure 13, we detected human action through five procedures. Figure 13a–c depicts the segmentation, which demonstrated good results.

**Figure 14:**
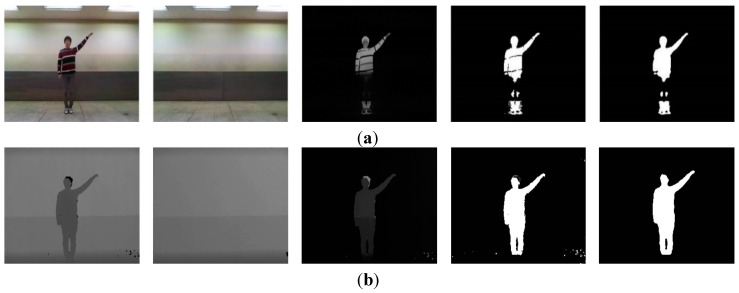
Difference of color and depth images in segmentation: (**a**) color-based segmentation; (**b**) depth-based segmentation.

Because depth information was used, we eliminated issues caused by variously colored clothing on the subjects. In the case of overlapping colors of background and clothing, it was difficult to accurately extract the subjects by a simple subtraction method. This challenge is illustrated in Figure 14. Because the subject’s pants appeared similar to the background color, loss of information in the subject’s leg region was evident.

When color information was used, we increased the threshold to minimize lighting and light reflection; we then detected the subject. If the threshold was lower, considerable noise occurred. Therefore, the background model was employed to apply color information. This was relatively slow and complex because the background model required a significant calculating and processing.

### 4.3. Feature Extraction

In our evaluation, features were extracted using DMH. However, the DM and DH methods each use MHI and HOG based on depth information. These methods can be compared to DMH as described below.

**Figure 15:**
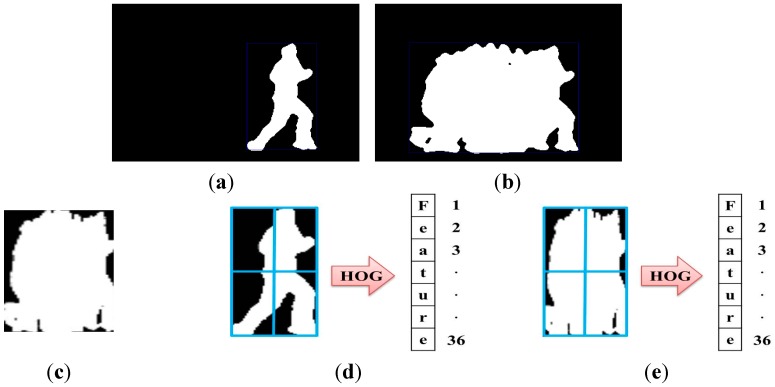
Segmentation images and feature extraction processes: (**a**) motion image; (**b**) MHI image; (**c**) DM; (**d**) DH; (**e**) DMH.

DM [43] offers the advantage of including previous information. However, it is difficult to simultaneously handle and requires many calculations because the received image has a greater amount of data than a feature vector. Additionally, its processing speed is slower than that of DMH. DH [44], on the other hand, extracts the feature vector for the action image at each moment, and its processing speed is faster than that of DMH. However, it includes no previous information; moreover, it has less information than DMH. Therefore, if a lot of information is required, DH should be added to the other method. DMH offers the advantages of the above two methods. For this reason, it is useful and accurately recognizes human action compared to other methods. In terms of processing speed, because MHI and HOG are fast, DMH does not notably affect recognition speed. These three methods are shown as Figure 15c–e. Figure 15a is a motion image, and Figure 15b is an image obtained by using MHI. Figure 15c–e, respectively, are DM, DH, and DMH. DH was obtained as Figure 15a; DMH was based on Figure 15b.

### 4.4. Action Modeling

When an action model was created, HMM was comprised of the L-R model structure based on an action sequence obtained through k-means clustering. In addition, action models were created according to the number of states in each of the actions. It depended on the complexity of the action and the number of states flexibly increased or decreased. On the other hand, action models created by a fixed state showed poor performance. Simple actions may have shown good performance in a small number of states; however, if an action used an unnecessarily large number of states, a complex structure was formed and affected calculation time.

**Figure 16:**
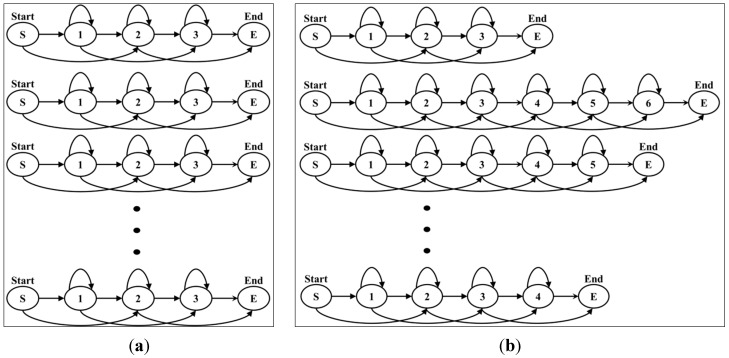
Example structure of fixed and flexible action models: (**a**) fixed action models; (**b**) flexible action models.

**Figure 17:**
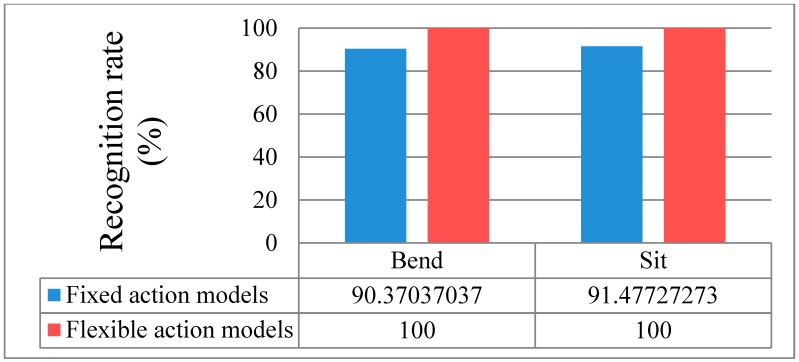
Comparison of fixed and flexible action models.

Figure 16a shows fixed action models with the same number of states, while Figure 16b shows flexible action models. In the case of fixed action models, the recognition rate was lower than that of flexible action models because complex actions are similar to simple actions. Examples of the comparison of forms of action models are shown in Figure 17, which indicates the recognition rates of Bend and Sit actions. 

The fixed action models had a 90.4% recognition rate (13 errors) for the Bend action and a 91.5% recognition rate (15 errors) for the Sit action. On the other hand, the flexible action models produced a 100% recognition result for two actions. The remaining results are outlined in the recognition discussion further below.

### 4.5. Action Spotting

**Figure 18:**
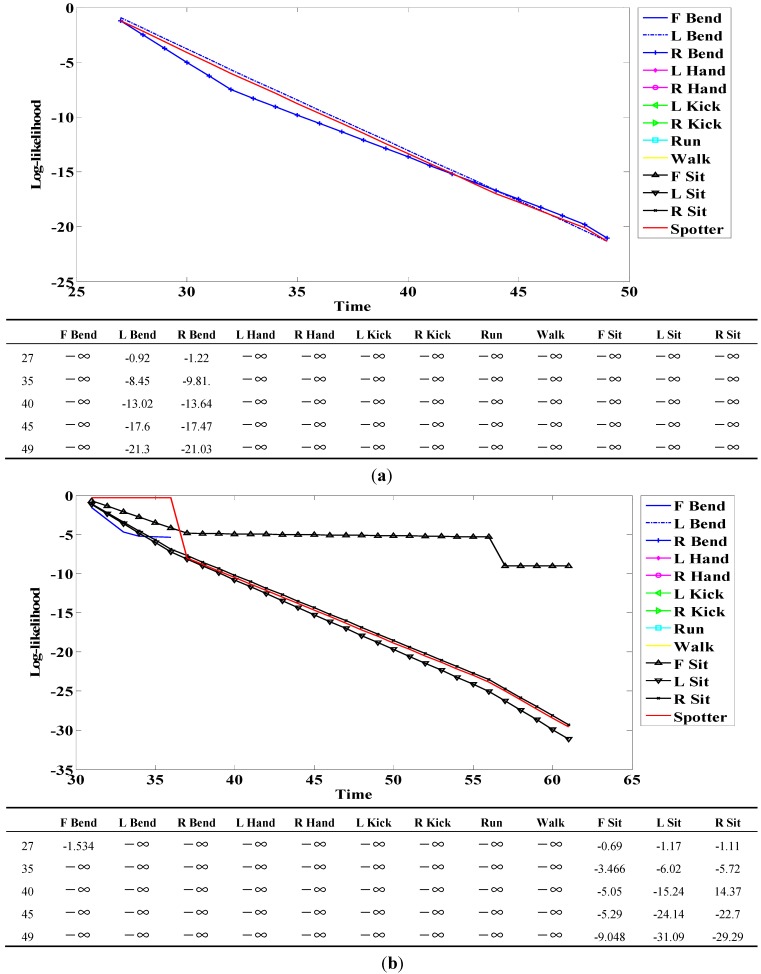
Example of log-likelihood for (**a**) R Bend; and (**b**) F Sit.

When a person engages in a continuous action, meaningless actions exist among meaningful ones. The spotter model filters meaningless actions and extracts meaningful actions; it is therefore useful for recognizing actions. The spotter model was created using HMM-like action models. The likelihood of action models representing meaningful action obtained by spotting was higher than that of spotter the model. We detected start and end points based on the spotter model and then recognized actions through corresponding sequences from start to end points. The following figures and tables show examples of the log-likelihood for corresponding sequences from start to end points.

Here, all log-likelihoods for the twelve actions are shown to explain certain cases; the six actions were described in the recognition discussion. In Figure 18, the log-likelihood results are indicated from the start point to the point corresponding to time. Similar actions generally had a log-likelihood and other actions commonly had negative infinity (−∞) because there was no correlation. The log-likelihood of L Bend was higher than that of R Bend, as shown in Figure 18a at the start (first) point; however, it gradually changed over time. As a result, the log-likelihood of R Bend was higher than that of L Bend at the end (last) point. As shown in Figure 18b, F Bend appeared to be similar to Sit in the early stage; however, the likelihood of F Bend had negative infinity (−∞) from the middle stage. Therefore, there was a correlation between Bend and Sit actions; however, each of the actions was predominantly recognized at the end point.

**Figure 19:**
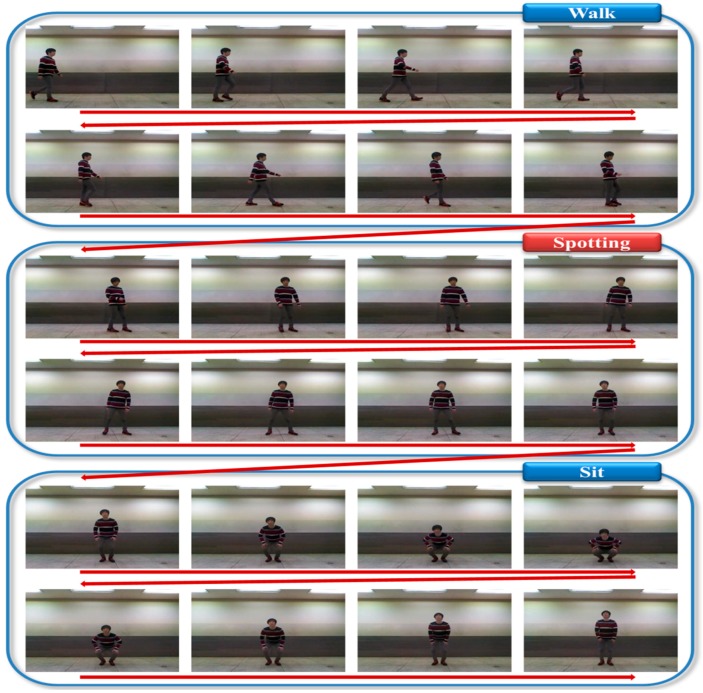
Experimental spotting of continuous action images.

Figure 19 is an example of filtering by the spotting method when meaningless action occurred between two meaningful actions. We connected images with an interval of four frames because many images could not be shown. Figure 19 illustrates a person sitting after walking.

The meaningless action between the meaningful actions (Walk and Sit) was filtered by the spotting method; the numbers spotted by the spotter model are outlined in Table 2.

**Table 2:** Spotter model experimental results.

	F Bend	L Bend	R Bend	L Hand	R Hand	L Kick	R Kick	Run	Walk	F Sit	L Sit	R Sit	Test
Actual	43	45	47	50	55	84	99	79	48	54	54	68	42
Spotter	43	45	47	50	55	84	99	79	48	54	54	68	41
Error	0	0	0	0	0	0	0	0	0	0	0	0	1

The total number of actions used in this experiment was 768. Compared to the spotter model, the proposed model produced a 99.9% success rate. During the evaluation, one error occurred; nevertheless, this result can be viewed as successful. We performed the recognition experiment based on this result.

### 4.6. Action Recognition

When an image frame was received, segmentation and feature extraction were performed. We then created a sequence corresponding to the start and end points detected by the spotting method. In the recognition process, the input sequence was applied to the action models; as the result, action was recognized according to likelihood.

**Figure 20:**
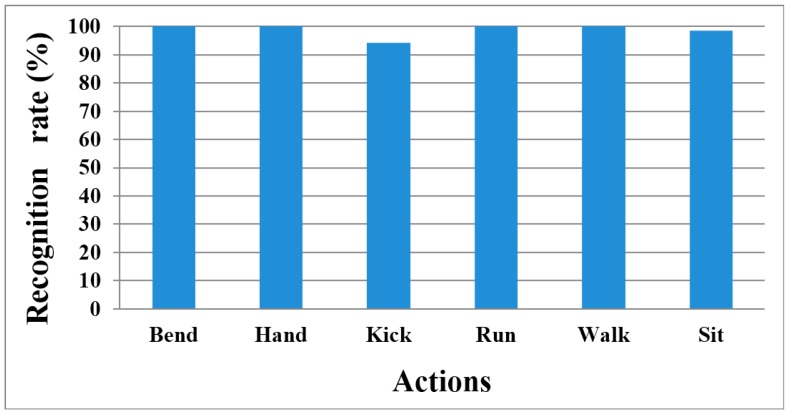
Recognition rate chart of all actions.

In our evaluation, we performed start and end point detection by using the spotter model instead of the sliding window. The proposed method was relatively fast and easy to perform, whereas the sliding window type was simple for constructing a window; however, window size affected recognition accuracy. Moreover, the smaller the window size, the more frequently HMM had to repeat the calculation. Therefore, we recognized continuous human action by using the proposed method. The recognition results are outlined as Table 3 and Figure 20.

In addition, we used the benchmarked dataset (WEIZMANN). This dataset is similar to the actions used in our experiment; however, it did not include continuous action and depth information. Nevertheless, our method showed 98.21% recognition accuracy with this dataset because we used action models that change the number of states depending on the complexity of the action.

**Table 3:** Confusion matrix for recognition results of all actions.

	Bend	Hand	Kick	Run	Walk	Sit
Bend	144	0	0	0	0	0
Hand	0	113	0	0	0	0
Kick	0	11	177	0	0	0
Run	0	0	0	80	0	0
Walk	0	0	0	0	53	0
Sit	3	0	0	0	0	187

## 5. Conclusions

In this paper, we have proposed methods for recognizing six everyday human actions (Bend, Sit, Raise Hand, Kick, Run, and Walk). Focusing on continuous human action recognition and spotting, these methods describe feature extraction by using DMH and the flexible action model according to complexity of action, and by using the spotter model for filtering meaningless action while spotting meaningful action.

The DMH feature is fast and effective because it includes previous information and easily and simply segments background and foreground. The number of states for action models is adjusted by complexity of action. Simple actions use a small number of states, while complex actions employ a large number of states. Recognition accuracy is increased by using flexible action models. Moreover, the spotter model is a simply connected structure. Start and end points are detected by comparing the likelihoods of the spotter model and action models. Start and end point detection extracts meaningful action and filters meaningless action for accuracy.

Our experiments showed that the proposed methods provided successful results. However, slight improvements are needed, or insufficient parts should be supplemented. In the future, it is necessary to add and test various actions to evaluate accurate performance. Therefore, we will use various data for subsequent experiments. If the results of these experiments are promising, our methods may be applied to important areas, such as surveillance [45], human-robot interactions [43,46], among others.
